# Supplementary material for: An integrated genomic regulatory network of virulence-related transcriptional factors in Pseudomonas aeruginosa
Source: Nat Commun. 2019 Jul 3;10:2931. doi: 10.1038/s41467-019-10778-w (PMC6610081; doi:10.1038/s41467-019-10778-w)
Supplement: Supplementary file 1 — Supplementary Information [file 41467_2019_10778_MOESM1_ESM.pdf]

Supplementary Information

An integrated genomic regulatory network of virulence-related transcriptional factors in *Pseudomonas aeruginosa*

Hao Huang1†, Xiaolong Shao2†, Yingpeng Xie1†, Tingting Wang1, Yingchao Zhang2, Xin Wang1*, Xin Deng1*

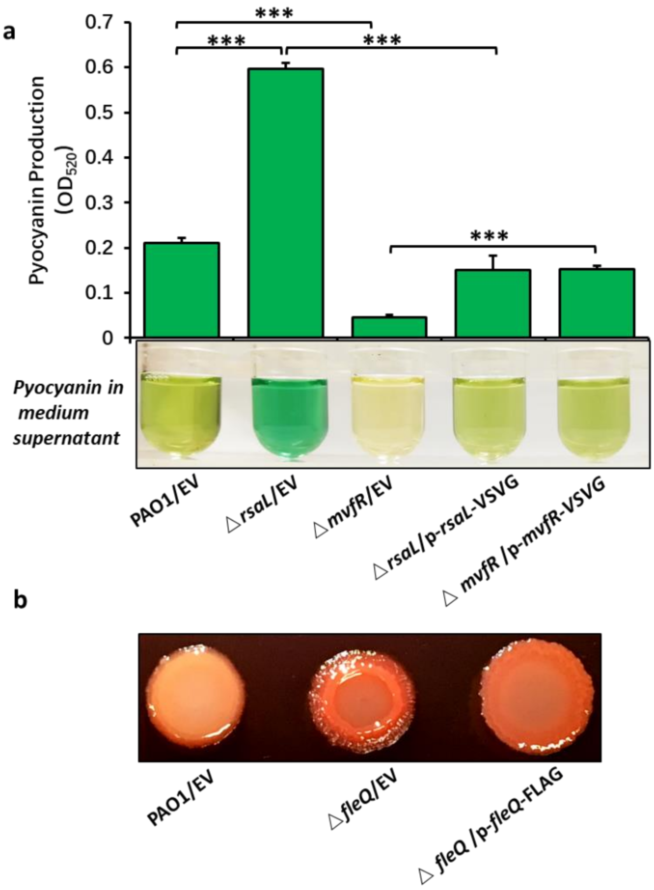

Supplementary Figure 1. The vsvG and FLAG-tag have no effect on the bioactivity of RsaL, MvfR and FleQ in *P. aeruginosa*. (a) The vsvG-tagged RsaL or MvfR functionally complement their corresponding deletion strains in a pyocyanin production assay[1, 2]. *$P < 0.05$, **$P < 0.01$ and ***$P < 0.001$. Error bars show standard deviations. **(b)** A Congo Red assay also showed that FLAG-tagged FleQ indeed complemented the colony morphology of △*fleQ* strain[3].

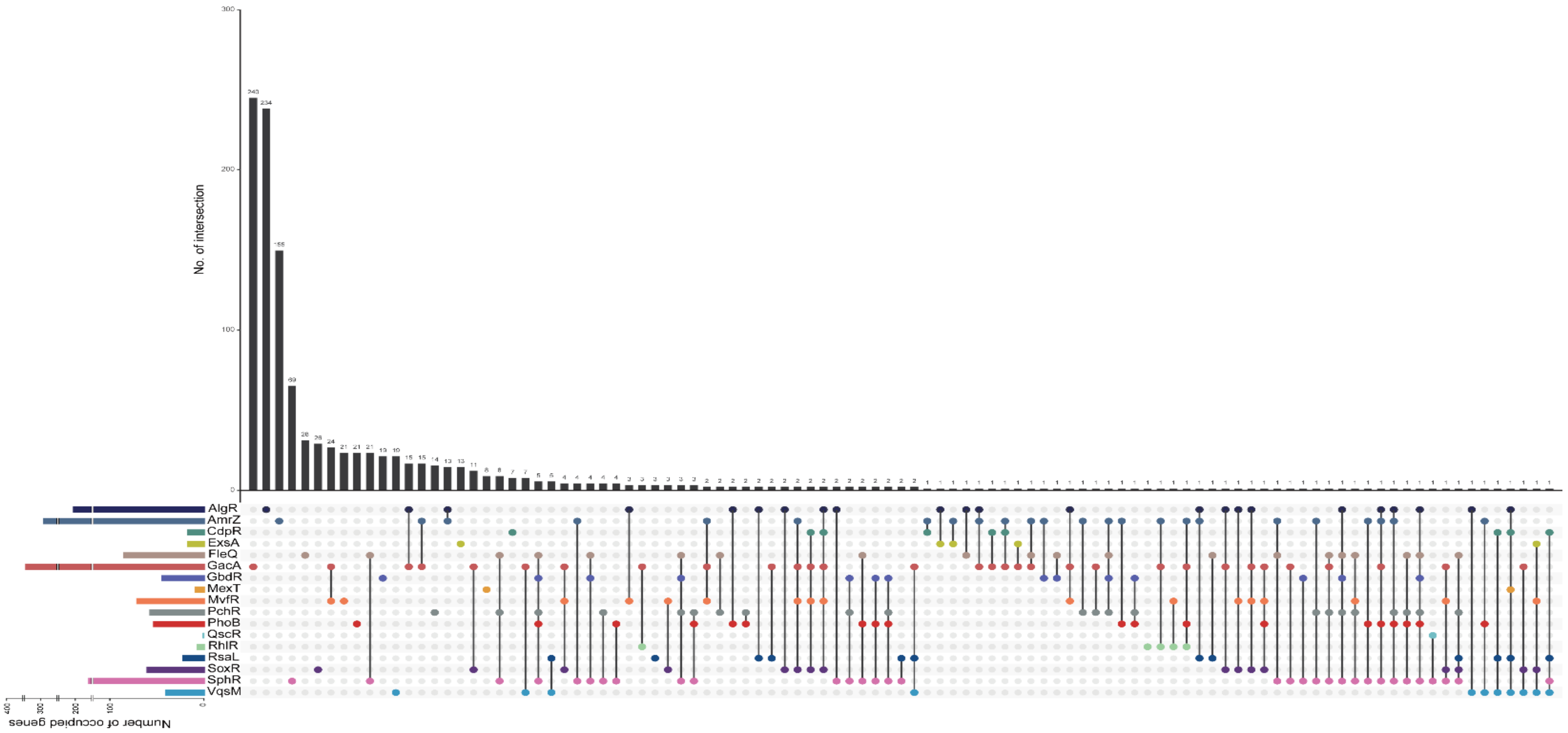

Supplementary Figure 2. The overlap of 20 TFs occupied genes based on the ChIP-seq data. The histogram in upper part represents the number of occupied genes of each TF. The lower part represents the overlap of different TFs target genes (TF-binding peaks located on promoters of genes), the histogram in right part represents the number of genes in individual/overlapped set.

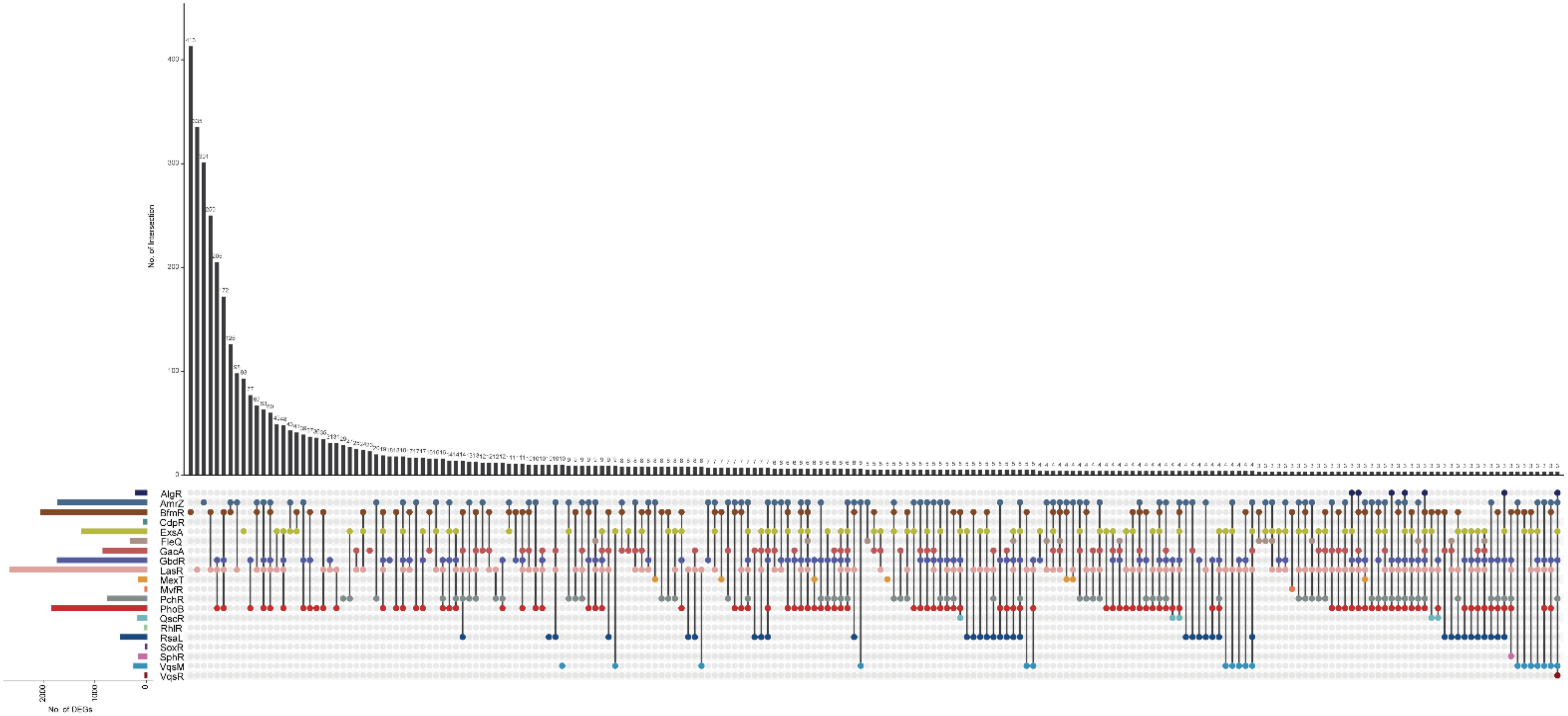

Supplementary Figure 3. The overlap of differential genes observed in 20 mutant TFs based on RNA-seq data. The histogram in upper part represents the number of differential genes in each mutant TF. The lower part represents the overlap of differential genes, the histogram in right part represents the number of genes in individual/overlapped (more than 3 genes) set.

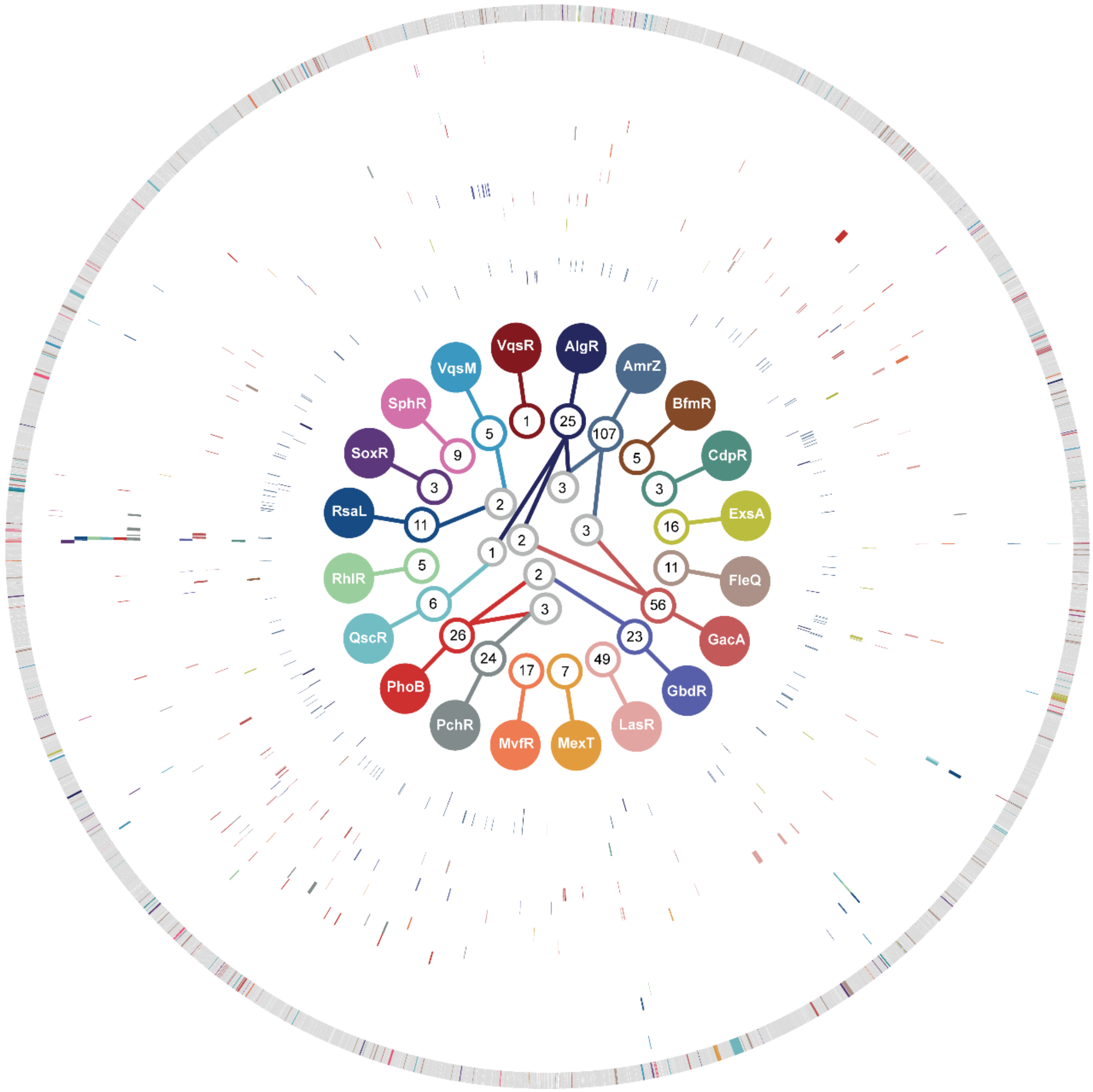

Supplementary Figure 4. The landscape of transcriptional factors regulome in *P. aeruginosa* chromosome. The outer circle summarizes the distribution of all 5,704 genes in *Pseudomonas aeruginosa* genome. These genes which can be regulated by multiple TFs are indicated in white. The inner twenty circles show the genes regulated by individual transcription factor. The innermost diagram represents the direct crosstalk between six most frequent transcriptional factors.

a

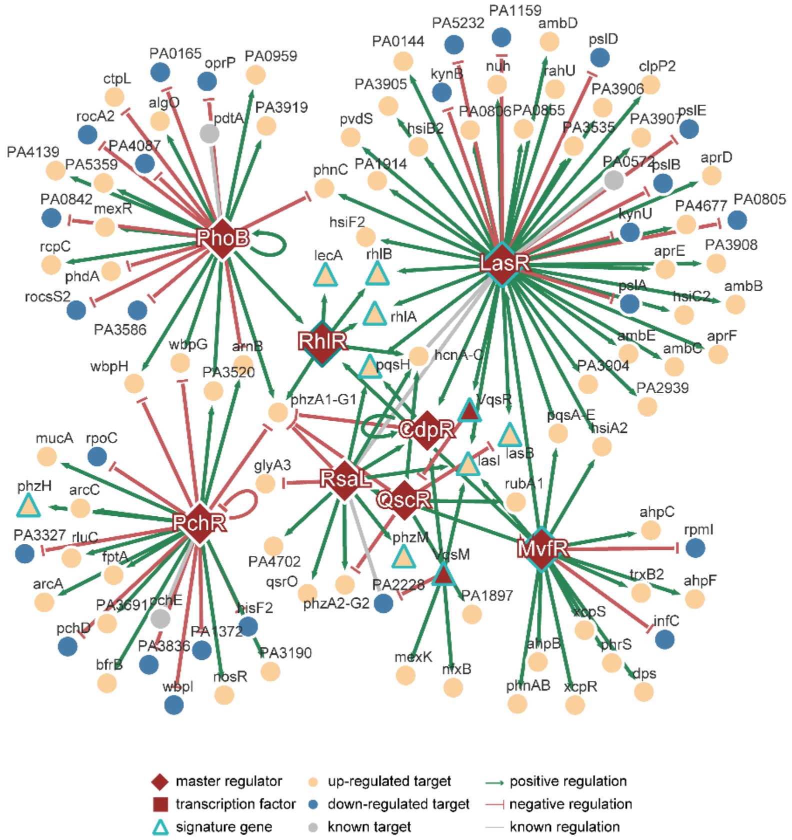

b

TF	No. of targets	Total No. of hits	Observed hits	*P*	BH-adjusted *P*
**RsaL**	25	34	17	1.49E-14	2.99E-13
**QscR**	20	34	15	9.12E-14	9.12E-13
**RhlR**	13	34	10	2.26E-09	1.51E-08
**CdpR**	9	34	8	1.67E-08	8.33E-08
**MvfR**	22	34	10	2.92E-06	1.17E-05
**PchR**	30	34	8	2.71E-03	9.03E-03
**PhoB**	32	34	8	4.26E-03	0.01
**LasR**	55	34	11	4.81E-03	0.01
GbdR	29	34	6	0.03	0.07
VqsM	5	34	1	0.37	0.69
BfmR	8	34	1	0.54	0.89
AlgR	25	34	2	0.68	1

Supplementary Figure 5. Network analysis identified RsaL, QscR, RhlR, MvfR, CdpR, PchR, PhoB and LasR as the master regulators of QS. (**a**) Visualization of master regulators of QS. TFs were shown as squares with different color, QS-associated genes were highlighted with frames. The red solid line means negative regulation and green solid line means positive regulation. LasR located in the central position of QS and directly regulates the expression level of 55 genes. (**b**) Master regulator analysis identified RsaL, QscR, RhlR, CdpR, MvfR, PchR, PhoB and LasR (bold font) as master regulators regulating QS in our network (Hypergeometric test, BH-adjusted $P < 0.05$).

a

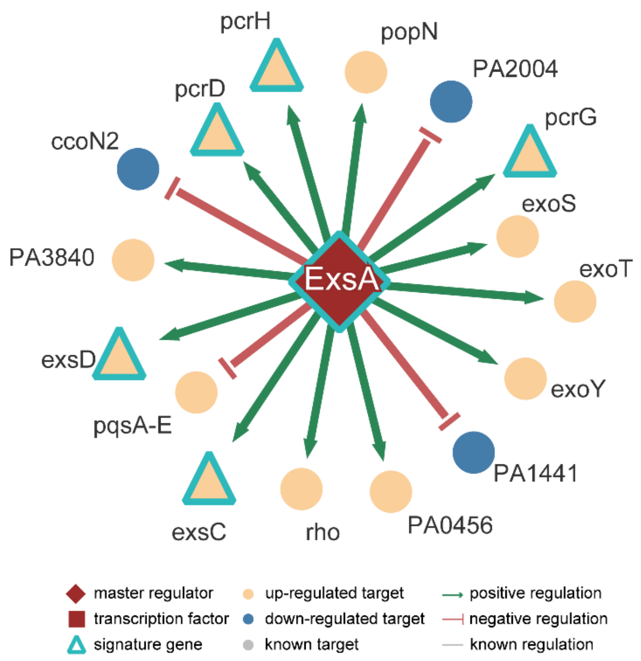

b

TF	No. of targets	Total No. of hits	Observed hits	*P*	BH-adjusted *P*
**ExsA**	20	7	5	4.99E-06	9.97E-05
VqsM	5	7	1	0.09	0.90
GacA	56	7	1	0.68	1

Supplementary Figure 6. Network analysis identified ExsA is the master regulator of T3SS. (**a**) Visualization of master regulators of T3SS. TFs were shown as squares with different color. The red solid line means negative regulation and green solid line means positive regulation. T3SS-associated are highlighted as rectangles. Seven targets genes involved in network are verified and show as positive result. ExsA directly regulated 20 genes, 5 of which are T3SS related genes. (**b**) Master regulator analysis identified ExsA (bold font) as mater regulator regulating T3SS in our network (Hypergeometric test, BH-adjusted $P < 0.05$).

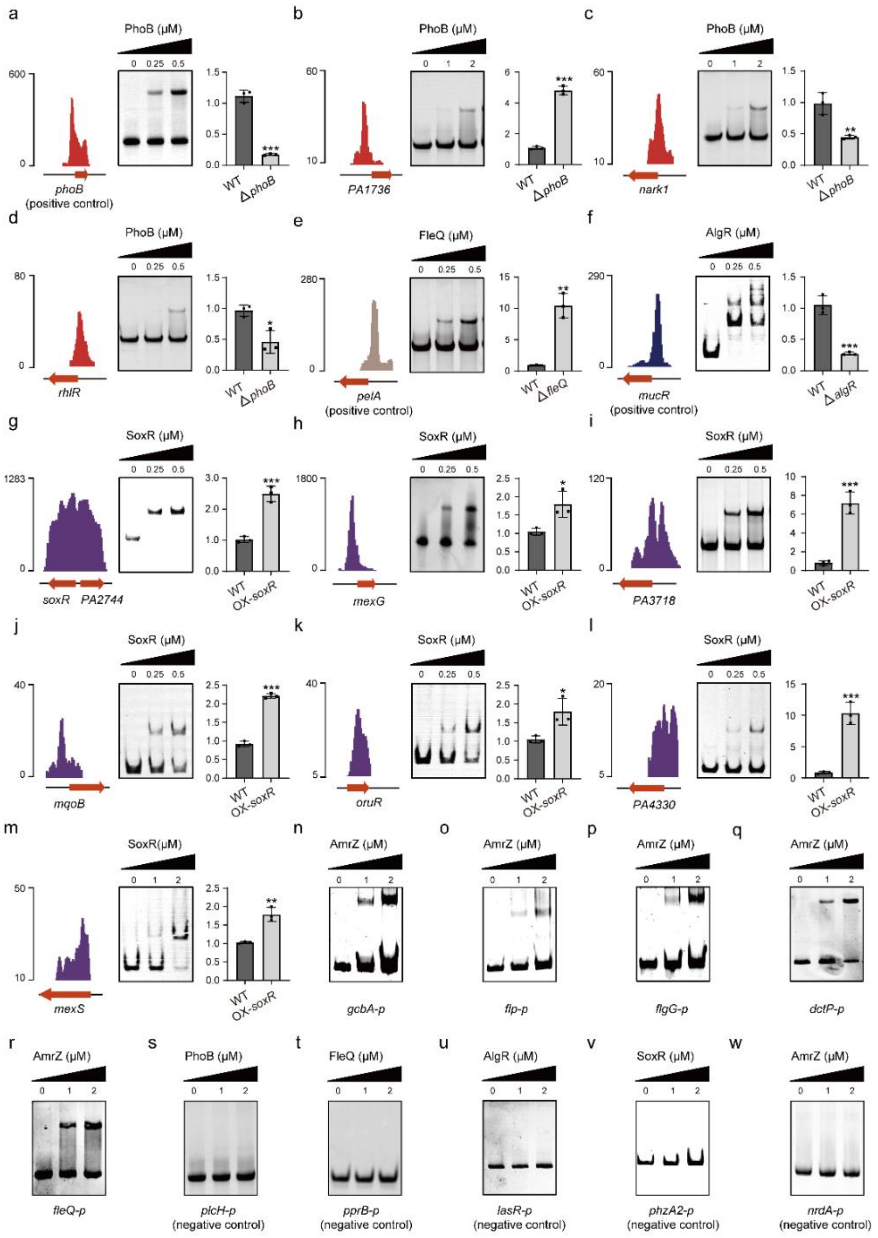

Supplementary Figure 7. EMSA and RT-qPCR verification of PhoB, FleQ, AlgR, SoxR and AmrZ. PhoB regulated the expression of *phoB* (positive control)*,* PA1736, *narK1* and *rhlR* (**a-d**). FleQ bound to the promoter of *pleA* and negatively regulated its expression (positive control) (**e**). AlgR bound to the promoter of mucR and positively regulated its expression (positive control) (**f**). SoxR regulated the expression of PA2744, *mexG*, PA3718, *mqoB, oruR*, PA4330 and *mexS* (**g-m**). AmrZ bound to the promoter of *gcbA, flp, flgG, dctP* and *fleQ* (**n-r**). PhoB, FleQ, AlgR, SoxR and AmrZ didn't bound to the promoter of *plcH, pprB, lasR, phzA2* and *nrdA*, respectively (**s-w**). The targets promoter regions were added to the reaction mixtures at 40 ng each well. Each protein

was added to reaction buffer in lanes with different gradient concentrations as shown in figures accordingly. For RT-qPCR, the relative expression of target genes in the wild-type PAO1 was set to 1, and the other values were adjusted accordingly. All experiments were independently repeated at least three times. Two-tailed Student's t-tests were used to examine the mean differences between the data groups. *$P < 0.05$, **$P < 0.01$ and ***$P < 0.001$. Error bars show standard deviations.

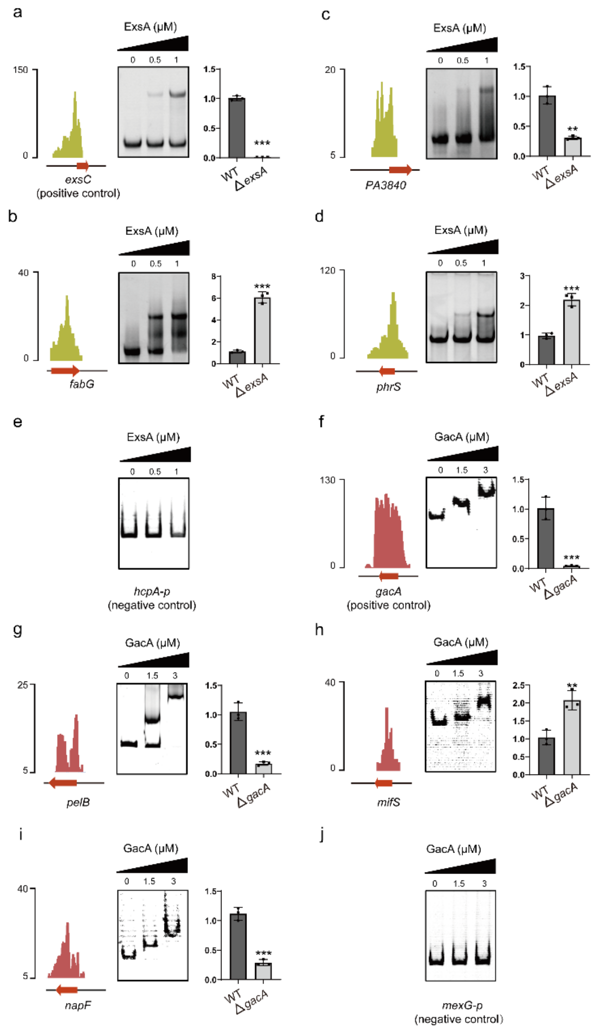

Supplementary Figure 8. ExsA and GacA directly regulated the expression of various functional genes. ExsA regulated the expression of *exsC,* PA3840, *fabG* and *phrS*, but didn't bind to the promoter of *hcpA* (**a-e**). GacA directly regulated the expression of *gacA pelB, mifS* and *napF*, but didn't bind to *mexG* promoter at the same protein concentration (**f-j**). PCR products containing these promoter regions were added to the reaction mixtures at 40 ng each well. Each protein was added to reaction buffer in lanes with 0, 0.5, 1.0 μM (for ExsA) or 0, 1.5, 3.0 μM (for GacA), respectively. For RT-qPCR, the relative expression of target genes in the wild-type PAO1 was set to 1, and the other values were adjusted accordingly. All experiments were independently repeated at least three times. Two-tailed Student's t-tests were used to examine the mean differences between the data groups. $*P < 0.05$, $**P < 0.01$ and $***P < 0.001$. Error bars show standard deviations.

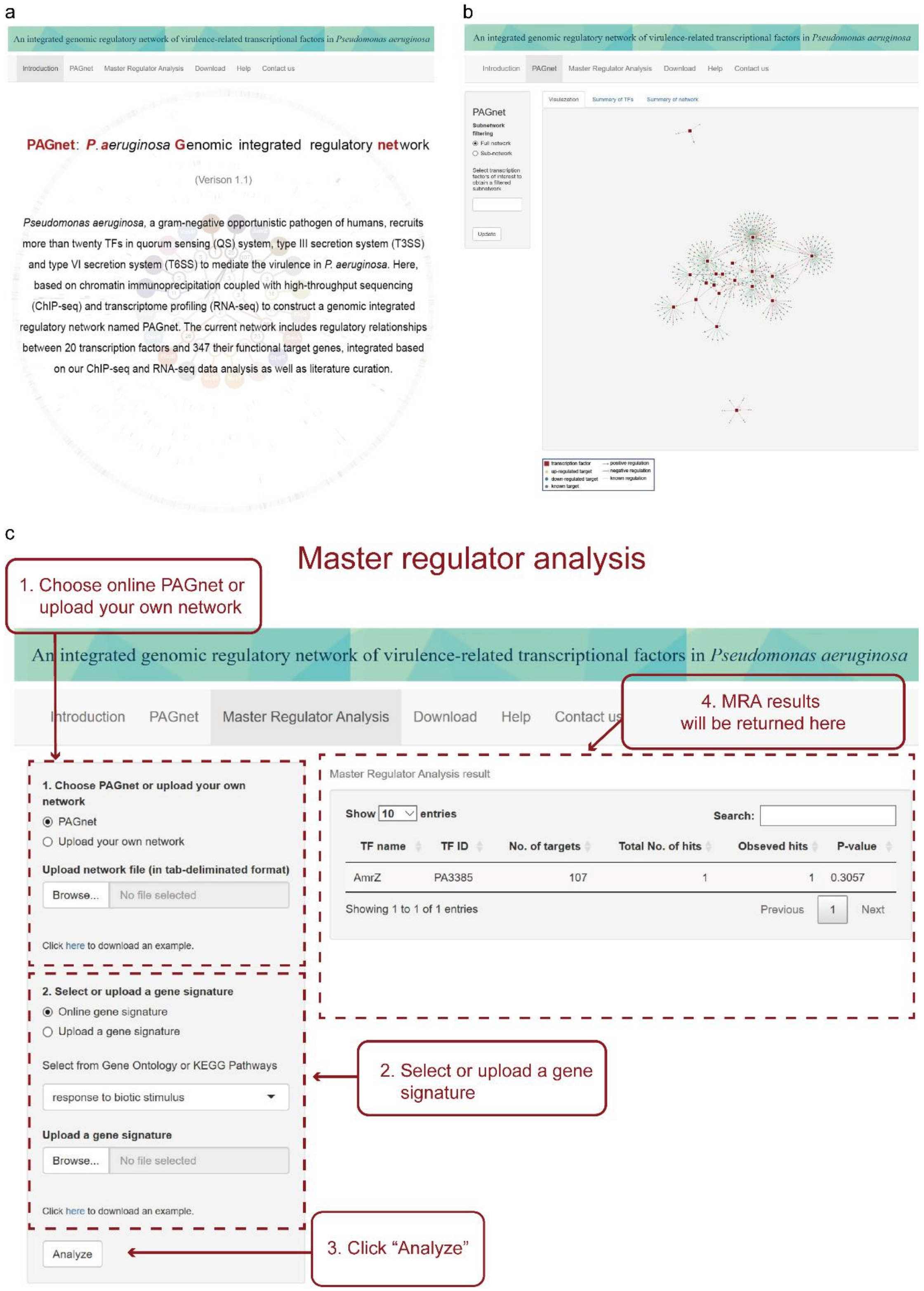

Supplementary Figure 9. Screenshots of PAGnet online platform. (**a**) Appearance of the homepage. (**b**) Online master regulator analysis functionality. A step-by-step guide was shown. (**c**) Master regulator analysis

Supplementary Table 1. The known functions of 20 regulators were summarised based on our study and other previous studies.

Transcription factors	Major known Functions
**AlgR**	AlgR positively regulates concentrations of intracellular c-di-GMP and then positively regulates biofilm production [4]. The phosphorylation status of AlgR controls swarming motility and rhamnolipid production [5]. AlgR Inhibits T3SS through regulateing RsmAYZ posttranscriptional regulatory system [6] .
**AmrZ**	AmrZ directly represses diguanylate cyclase-encoding gene *adcA,* and then leads to reduced amounts of the second messenger c-di-GMP, which finally result in biofilm dissolution [7]. AmrZ is a global T6SS transcriptional regulator [8].
**BfmR**	BfmR is a biofilm maturation regulator [9, 10]. BfmR negatively controls the *rhl* QS system in direct manner [11].
**CdpR**	CdpR negatively modulates bacterial virulence. CdpR is negatively autoregulated and be able to directly binds to *pqsH-cdpR* intergenic region and regulates *pqsH* expression [12].
**ExsA**	ExsA regulates the expression of more than 40 T3SS genes [13].
**GacA**	GacA positively regulates the transcription of RsmZ/Y, and further block the repression effects of RsmA [14].
**LasR**	QS regulator LasR controls the expression of more than 300 genes. It activates the level of c-di-GMP by binding to *tbpA* promoter and activates PQS system by binding to *pqsR* promoter [15, 16, 17].
**MexT**	MexT downregulates several virulence determinants, such as T3SS gene expression, pyocyanin biosynthesis and early surface attachment [18].
**MvfR**	MvfR contributes to the activation of both RhlR and LasR QS systems and also induces type 2 and type 6 protein secretion systems [19].
**QscR**	In the absence of AHL, QscR can multimerize and form heterodimers with LasR and RhlR, and further lead to negative effects on the LasR and RhlR target genes [20].
**RhlR**	RhlR regulates the expression of genes involved in biofilm formation and also other genes encoding virulence factors[21]. RhlR represses the PQS pathway by binding to *pqsR* promoter [16].
**RsaL**	RsaL activates the expression of *pqsH* and further positively regulates biofilm formation, at the same time, RsaL activates the expression of *cdpR* and then suppresses the biosynthesis of pyocyanin [1].
**SoxR**	Generally, activation of SoxR is resulted from increased levels of O2.- and NO in host environments. However, SoxR is required for virulence, but not a key player in the oxidative stress response or in antibiotic resistance [22].
**VqsM**	VqsM directly binds to the lasI promoter region and regulates the expression of lasI, while indirectly regulating the Rhl system. In addition, VqsM directly binds to exsA promoter and controls the T3SS and have a negative regulation of antibiotic resistance [23].
**VqsR**	VqsR negatively regulates qscR expression and positively controls several phenotypes, such as pyocyanin, motility, and colony morphology [24].
**PhoB**	Bacterial-type flagellum-dependent swarming motility [24], positive regulation of cellular response to phosphate starvation [25]and phosphorelay response regulator activity [26].

**GbdR**	Regulation of transcription, DNA-templated [27], glycine betaine catabolic process [27] and positive regulation of phosphatase activity [28].
**PchR**	Regulation of siderophore pyochelin [29], ferripyochelin receptor gene (*fptA*) expression [30].
**SphR**	SphR is a sphingosine-responsive regulator and sphingolipid binding activity [31].
**FleQ**	Regulation of bacterial-type flagellum-dependent cell motility [32, 33], cyclic-di-GMP binding activity and biofilm formation [3, 34], positive regulation of cell adhesion [32] and positive regulation of cilium-dependent cell motility [35].

Supplementary Table 2. Experimental conditions for each of the ChIP-seq and RNA-seq transcription factors used in this study. The major biological pathway for each transcription factor is shown. Our samples were normalised at the mid-log phase (OD_{600} = 0.6) in the LB medium. Data from other studies are cited accordingly.

No.	TFs	ChIP-seq	Directly binding genes	Condition	References	RNA-seq	Differentially expressed genes	Condition	References
1	AlgR	Yes, PAO1	289	LB, OD_{600}=0.6	This study	Yes, PAO1	201	LB, OD_{600}=0.6	This study
2	AmrZ	Yes, PAO1	209	LB, OD_{600}=0.6	[7]	Yes, PAO1	1708	LB, OD_{600}=0.6	[7]
3	BfmR	No, PAO1	No, ChIP-cloning	24-hr-biofilms	[10]	PAO1, Microarray	2042	1/20 diluted LB or VBMM medium (citrate and arabinose), 22°C	[10]
4	CdpR	Yes, PAO1	17	LB, OD_{600}=0.6	[12]	Yes, PAO1	47	LB, OD600=0.6	This study
5	ExsA	Yes, PAO1	17	LB, OD_{600}=0.6	This study	Yes, PAO1	1249	LB, OD_{600}=0.6 + EGTA	This study
6	GacA	Yes, PAO1	348	LB, OD_{600}=0.6	This study	Yes, PAO1	833	LB, OD_{600}=0.6	This study
7	LasR	No, PAO1, ChIP *in vitro*	73	LB, OD_{600}=0.6	[15]	Yes, PAO1	2642	LB, OD_{600}=0.6	This study
8	MexT	Yes, PAO1	9	LB, OD_{600}=0.6	This study	Yes, PAO1	143	LB, OD_{600}=0.6	[18]
9	MvfR	Yes, PAO1	71	LB, OD_{600}=0.6	This study	Yes, PAO1	20	LB, OD_{600}=0.6	This study
10	QscR	Yes, PAO1	1	LB, OD_{600}=0.6	This study	Yes, PAO1	202	LB, OD_{600}=0.6	This study
11	RhlR	Yes, PAO1	7	LB, OD_{600}=0.6	This study	Yes, PAO1	23	LB, OD_{600}=0.6	This study
12	RsaL	Yes, PAO1	22	LB, OD_{600}=0.6	[1]	Yes, PAO1	495	LB, OD_{600}=0.6	This study
13	SoxR	Yes, PAO1	60	LB, OD_{600}=0.6	This study	PAO1, Microarray	6	TSB, OD600=0.5, H_2O_2/PQ	[22]
14	VqsM	Yes, PAO1	40	LB, OD_{600}=0.6	[23]	Yes, PAO1	63	LB, OD_{600}=0.6	This study
15	VqsR	PAO1, Genes containing inverted repeat sequences	51	LB, OD_{600}=0.6	[24]	Yes, PAO1	21	LB, OD_{600}=0.6	This study

16	PhoB	Yes, PAO1	53	LB, OD_{600}=0.6	This study	Yes, PAO1	1828	LB, OD_{600}=0.6	This study
17	GbdR	Yes, PAO1	44	LB, OD_{600}=0.6	This study	Yes, PAO1	1722	LB, OD_{600}=0.6	This study
18	PchR	Yes, PAO1	57	LB, OD_{600}=0.6	This study	Yes, PAO1	741	LB, OD_{600}=0.6	This study
19	SphR	Yes, PAO1	153	LB, OD_{600}=0.6	This study	Yes, PAO1	140	LB, OD_{600}=0.6	This study
20	FleQ	Yes, PAO1	85	LB, OD_{600}=0.6	This study	Yes, PAO1	301	LB, OD_{600}=0.6	This study

Table S3. Comparison between the RNA-seq data in this study and the previous studies.

	RNA-seq in this study		RNA-seq or microarray in previous study		
TFs	Total number	Strains and cultural conditions	Total number	Strains and cultural conditions	References
AlgR	201	PAO1, RNA-seq, OD_{600} =0.6	47	PAO1, microarray, OD_{600} =0.4/0.6	[36]
ExsA	1249	PAO1, RNA-seq, OD_{600} =0.6	31	*P. aeruginosa* PAK, microarray, TSB (EGTA), OD_{600} =0.5	[37]
GacA	833	PAO1, RNA-seq, OD_{600} =0.6	772	*P. aeruginosa* M18, microarray, KMB, OD_{600}=5.0, 28°C	[38]
MvfR	20	PAO1, RNA-seq, OD_{600} =0.6	133	PA14, microarray, LB, OD_{600}=1.5, 2.5 and 3.5/PA14, LB, OD600=2 and 4	[16, 19]
RsaL	495	PAO1, RNA-seq, OD_{600} =0.6	341	PAO1, microarray, LB, OD_{600}=2.0	[39]
VqsM	63	PAO1, RNA-seq, OD_{600} =0.6	240	PAO1, Microarray, LB, OD_{600}=1.5	[40]
VqsR	21	PAO1, RNA-seq, OD_{600} =0.6	131	*P. aeruginosa* TB*,* microarray, ABC minimal medium (Sodium citrate) OD_{600}=1.0	[41]
PhoB	1828	PAO1, RNA-seq, OD_{600} =0.6	76	PA14, RNA-seq, DeMoss or M9 medium (low/high phosphate)	[42]
GbdR	1722	PAO1, RNA-seq, OD_{600} =0.6	137	PA14, microarray, MOPS minimal medium (pyruvate, choline or choline analogue), OD_{600}=0.45	[43]
SphR	140	PAO1, RNA-seq, OD_{600} =0.6	94	PAO1, microarray, PY medium (sodium taurodeoxycholate, SM), 30 ℃, 8 hr	[44]

Supplementary References

1. Kang H*, et al.* Crystal structure of *Pseudomonas aeruginosa* RsaL bound to promoter DNA reaffirms its role as a global regulator involved in quorum-sensing. *Nucleic Acids Res* **45**, 699-710 (2017).

2. Zaborina O*, et al.* Dynorphin activates quorum sensing quinolone signaling in *Pseudomonas aeruginosa*. *PLoS Pathog* **3**, e35 (2007).

3. Hickman JW, Harwood CS. Identification of FleQ from *Pseudomonas aeruginosa* as a c-di-GMP-responsive transcription factor. *Mol Microbiol* **69**, 376-389 (2008).

4. Kong W*, et al.* ChIP-seq reveals the global regulator AlgR mediating cyclic di-GMP synthesis in *Pseudomonas aeruginosa*. *Nucleic Acids Res* **43**, 8268-8282 (2015).

5. Okkotsu Y, Tieku P, Fitzsimmons LF, Churchill ME, Schurr MJ. *Pseudomonas aeruginosa* AlgR phosphorylation modulates rhamnolipid production and motility. *J Bacteriol* **195**, 5499-5515 (2013).

6. Intile PJ, Diaz MR, Urbanowski ML, Wolfgang MC, Yahr TL. The AlgZR two-component system recalibrates the RsmAYZ posttranscriptional regulatory system to inhibit expression of the *Pseudomonas aeruginosa* type III secretion system. *J Bacteriol* **196**, 357-366 (2014).

7. Jones CJ*, et al.* ChIP-Seq and RNA-Seq reveal an AmrZ-mediated mechanism for cyclic di-GMP synthesis and biofilm development by *Pseudomonas aeruginosa*. *PLoS Pathog* **10**, e1003984 (2014).

8. Allsopp LP*, et al.* RsmA and AmrZ orchestrate the assembly of all three type VI secretion systems in *Pseudomonas aeruginosa*. *Proc Natl Acad Sci U S A* **114**, 7707-7712 (2017).

9. Petrova OE, Sauer K. A novel signaling network essential for regulating *Pseudomonas aeruginosa* biofilm development. *PLoS Pathog* **5**, e1000668 (2009).

10. Petrova OE, Schurr JR, Schurr MJ, Sauer K. The novel *Pseudomonas aeruginosa* two-component regulator BfmR controls bacteriophage-mediated lysis and DNA release during biofilm development through PhdA. *Mol Microbiol* **81**, 767-783 (2011).

11. Cao Q*, et al.* A novel signal transduction pathway that modulates *rhl* quorum sensing and bacterial virulence in *Pseudomonas aeruginosa*. *PLoS Pathog* **10**, e1004340 (2014).

12. Zhao J*, et al.* Structural and Molecular Mechanism of CdpR Involved in Quorum-Sensing and Bacterial Virulence in *Pseudomonas aeruginosa*. *PLoS Biol* **14**, e1002449 (2016).

13. Hovey AK, Frank DW. Analyses of the DNA-binding and transcriptional activation properties of ExsA, the transcriptional activator of the *Pseudomonas aeruginosa* exoenzyme S regulon. *J Bacteriol* **177**, 4427-4436 (1995).

14. Brencic A*, et al.* The GacS/GacA signal transduction system of *Pseudomonas aeruginosa* acts exclusively through its control over the transcription of the RsmY and RsmZ regulatory small RNAs. *Mol Microbiol* **73**, 434-445 (2009).

15. Gilbert KB, Kim TH, Gupta R, Greenberg EP, Schuster M. Global position analysis of the *Pseudomonas aeruginosa* quorum-sensing transcription factor LasR. *Mol Microbiol* **73**, 1072-1085 (2009).

16. Deziel E*, et al.* The contribution of MvfR to *Pseudomonas aeruginosa* pathogenesis and quorum sensing circuitry regulation: multiple quorum sensing-regulated genes are modulated without affecting *lasRI*, *rhlRI* or the production of N-acyl-L-homoserine lactones. *Mol Microbiol* **55**, 998-1014 (2005).

17. Ueda A, Wood TK. Connecting quorum sensing, c-di-GMP, *pel* polysaccharide, and biofilm formation in *Pseudomonas aeruginosa* through

tyrosine phosphatase TpbA (PA3885). *PLoS Pathog* **5**, e1000483 (2009).

18. Tian ZX, Fargier E, Mac Aogain M, Adams C, Wang YP, O'Gara F. Transcriptome profiling defines a novel regulon modulated by the LysR-type transcriptional regulator MexT in *Pseudomonas aeruginosa*. *Nucleic Acids Res* **37**, 7546-7559 (2009).

19. Maura D, Hazan R, Kitao T, Ballok AE, Rahme LG. Evidence for Direct Control of Virulence and Defense Gene Circuits by the *Pseudomonas aeruginosa* Quorum Sensing Regulator, MvfR. *Sci Rep* **6**, 34083 (2016).

20. Ledgham F, Ventre I, Soscia C, Foglino M, Sturgis JN, Lazdunski A. Interactions of the quorum sensing regulator QscR: interaction with itself and the other regulators of *Pseudomonas aeruginosa* LasR and RhlR. *Mol Microbiol* **48**, 199-210 (2003).

21. Mukherjee S, Moustafa D, Smith CD, Goldberg JB, Bassler BL. The RhlR quorum-sensing receptor controls *Pseudomonas aeruginosa* pathogenesis and biofilm development independently of its canonical homoserine lactone autoinducer. *PLoS Pathog* **13**, e1006504 (2017).

22. Palma M*, et al. Pseudomonas aeruginosa* SoxR does not conform to the archetypal paradigm for SoxR-dependent regulation of the bacterial oxidative stress adaptive response. *Infect Immun* **73**, 2958-2966 (2005).

23. Liang H, Deng X, Li X, Ye Y, Wu M. Molecular mechanisms of master regulator VqsM mediating quorum-sensing and antibiotic resistance in Pseudomonas aeruginosa. *Nucleic Acids Res* **42**, 10307-10320 (2014).

24. Liang H, *et al.* The *Pseudomonas aeruginosa* global regulator VqsR directly inhibits QscR to control quorum-sensing and virulence gene expression. *J Bacteriol* **194**, 3098-3108 (2012).

25. Faure LM, Llamas MA, Bastiaansen KC, de Bentzmann S, Bigot S. Phosphate starvation relayed by PhoB activates the expression of the *Pseudomonas aeruginosa* sigmavreI ECF factor and its target genes. *Microbiology* **159**, 1315-1327 (2013).

26. Monds RD, Silby MW, Mahanty HK. Expression of the Pho regulon negatively regulates biofilm formation by Pseudomonas aureofaciens PA147-2. *Mol Microbiol* **42**, 415-426 (2001).

27. Wargo MJ, Szwergold BS, Hogan DA. Identification of two gene clusters and a transcriptional regulator required for *Pseudomonas aeruginosa* glycine betaine catabolism. *J Bacteriol* **190**, 2690-2699 (2008).

28. Wargo MJ, Ho TC, Gross MJ, Whittaker LA, Hogan DA. GbdR regulates *Pseudomonas aeruginosa* plcH and pchP transcription in response to choline catabolites. *Infect Immun* **77**, 1103-1111 (2009).

29. Michel L, Gonzalez N, Jagdeep S, Nguyen-Ngoc T, Reimmann C. PchR-box recognition by the AraC-type regulator PchR of *Pseudomonas aeruginosa* requires the siderophore pyochelin as an effector. *Mol Microbiol* **58**, 495-509 (2005).

30. Heinrichs DE, Poole K. PchR, a regulator of ferripyochelin receptor gene (fptA) expression in *Pseudomonas aeruginosa*, functions both as an activator and as a repressor. *J Bacteriol* **178**, 2586-2592 (1996).

31. LaBauve AE, Wargo MJ. Detection of host-derived sphingosine by *Pseudomonas aeruginosa* is important for survival in the murine lung. *PLoS Pathog* **10**, e1003889 (2014).

32. Arora SK, Ritchings BW, Almira EC, Lory S, Ramphal R. A transcriptional activator, FleQ, regulates mucin adhesion and flagellar gene expression in *Pseudomonas aeruginosa* in a cascade manner. *J Bacteriol* **179**, 5574-5581 (1997).

33. Dasgupta N*, et al.* A four-tiered transcriptional regulatory circuit controls flagellar biogenesis in *Pseudomonas aeruginosa*. *Mol Microbiol* **50**, 809-824 (2003).

34. Baraquet C, Harwood CS. Cyclic diguanosine monophosphate represses bacterial flagella synthesis by interacting with the Walker A motif of the enhancer-binding protein FleQ. *Proc Natl Acad Sci U S A* **110**, 18478-18483 (2013).

35. Jyot J, Dasgupta N, Ramphal R. FleQ, the major flagellar gene regulator in *Pseudomonas aeruginosa*, binds to enhancer sites located either upstream or atypically downstream of the RpoN binding site. *J Bacteriol* **184**, 5251-5260 (2002).

36. Lizewski SE, *et al.* Identification of AlgR-regulated genes in *Pseudomonas aeruginosa* by use of microarray analysis. *J Bacteriol* **186**, 5672-5684 (2004).

37. Wolfgang MC, Lee VT, Gilmore ME, Lory S. Coordinate regulation of bacterial virulence genes by a novel adenylate cyclase-dependent signaling pathway. *Dev Cell* **4**, 253-263 (2003).

38. Wei X, Huang X, Tang L, Wu D, Xu Y. Global control of GacA in secondary metabolism, primary metabolism, secretion systems, and motility in the rhizobacterium *Pseudomonas aeruginosa* M18. *J Bacteriol* **195**, 3387-3400 (2013).

39. Rampioni G, Schuster M, Greenberg EP, Zennaro E, Leoni L. Contribution of the RsaL global regulator to *Pseudomonas aeruginosa* virulence and biofilm formation. *FEMS Microbiol Lett* **301**, 210-217 (2009).

40. Gribaldo L*, et al.* Acute toxicity. *Altern Lab Anim* **33 Suppl 1**, 27-34 (2005).

41. Juhas M, Wiehlmann L, Salunkhe P, Lauber J, Buer J, Tummler B. GeneChip expression analysis of the VqsR regulon of *Pseudomonas aeruginosa* TB. *FEMS Microbiol Lett* **242**, 287-295 (2005).

42. Bielecki P*, et al.* Cross talk between the response regulators PhoB and TctD allows for the integration of diverse environmental signals in *Pseudomonas aeruginosa*. *Nucleic Acids Res* **43**, 6413-6425 (2015).

43. Hampel KJ, LaBauve AE, Meadows JA, Fitzsimmons LF, Nock AM, Wargo MJ. Characterization of the GbdR regulon in *Pseudomonas aeruginosa*. *J Bacteriol* **196**, 7-15 (2014).

44. Okino N, Ito M. Molecular mechanism for sphingosine-induced *Pseudomonas* ceramidase expression through the transcriptional regulator SphR. *Sci Rep* **6**, 38797 (2016).
