# Supplementary material for: An integrated genomic regulatory network of virulence-related transcriptional factors in Pseudomonas aeruginosa
Source: Nat Commun. 2019 Jul 3;10:2931. doi: 10.1038/s41467-019-10778-w (PMC6610081; doi:10.1038/s41467-019-10778-w)
Supplement: Supplementary file 3 — Description of Additional Supplementary Files [file 41467_2019_10778_MOESM3_ESM.pdf]

Description of Additional Supplementary Files

File Name: Supplementary Data 1
Description: **Co-occurrence of multiple TFs revealed by ChIP-seq.** Genes were co-bound by at least two transcription factors are shown with the statistical significance of the co-occurrence (co-binding) of multiple TFs on the same promoter of the gene.

File Name: Supplementary Data 2
Description: **Co-occurrence of differentially expressed genes observed in multiple TFs mutant revealed by RNA-seq.** Genes were co-regulated by at least two transcription factors are shown with the statistical significance of co-occurrence of differential gene expression observed in multiple mutant TFs.

File Name: Supplementary Data 3
Description: **Genes functionally co-regulated by multiple TFs (PAGnet) based on ChIP-seq and RNA-seq.** Genes functionally co-regulated (intersection of Supplementary Data 1 and Supplementary Data 2) by at least two transcription factors are shown with the statistical significance.

File Name: Supplementary Data 4
Description: **Function enrichment of regulons in PAGnet.** Hypergeometric tests ($P < 0.05$) were performed for each regulon, which is based on functional gene sets from PseudoCAP including Gene Ontology and KEGG databases.

File Name: Supplementary Data 5
Description: **Bacterial strains, plasmids, and primers used in this study.**
