# Supplementary material for: An integrated genomic regulatory network of virulence-related transcriptional factors in Pseudomonas aeruginosa
Source: Nat Commun. 2019 Jul 3;10:2931. doi: 10.1038/s41467-019-10778-w (PMC6610081; doi:10.1038/s41467-019-10778-w)
Supplement: Supplementary file 9 — Reporting Summary [file 41467_2019_10778_MOESM9_ESM.pdf]

nature research

Corresponding author(s): Xin Deng

Last updated by author(s): May 17, 2019

Reporting Summary

Nature Research wishes to improve the reproducibility of the work that we publish. This form provides structure for consistency and transparency in reporting. For further information on Nature Research policies, see Authors & Referees and the Editorial Policy Checklist.

Statistics

For all statistical analyses, confirm that the following items are present in the figure legend, table legend, main text, or Methods section.

n/a	Confirmed	
☒	☐	The exact sample size (n) for each experimental group/condition, given as a discrete number and unit of measurement
☒	☐	A statement on whether measurements were taken from distinct samples or whether the same sample was measured repeatedly
☐	☒	The statistical test(s) used AND whether they are one- or two-sided<br>*Only common tests should be described solely by name; describe more complex techniques in the Methods section.*
☒	☐	A description of all covariates tested
☒	☐	A description of any assumptions or corrections, such as tests of normality and adjustment for multiple comparisons
☒	☐	A full description of the statistical parameters including central tendency (e.g. means) or other basic estimates (e.g. regression coefficient) AND variation (e.g. standard deviation) or associated estimates of uncertainty (e.g. confidence intervals)
☒	☐	For null hypothesis testing, the test statistic (e.g. F, t, r) with confidence intervals, effect sizes, degrees of freedom and P value noted<br>*Give P values as exact values whenever suitable.*
☒	☐	For Bayesian analysis, information on the choice of priors and Markov chain Monte Carlo settings
☒	☐	For hierarchical and complex designs, identification of the appropriate level for tests and full reporting of outcomes
☒	☐	Estimates of effect sizes (e.g. Cohen's d, Pearson's r), indicating how they were calculated

Our web collection on statistics for biologists contains articles on many of the points above.

Software and code

Policy information about availability of computer code

Data collection	No software was used.
Data analysis	1. ChIP-seq analysis<br>ChIP-seq reads were mapped to the P. aeruginosa genomes (NC_002516) using Bowtie (Version 1.2.1.1). All the experiments have two repeats and repeats were merged together for downstream analysis. Only the uniquely mapped reads were kept for the subsequent analyses. Binding peaks (P < 1e-5) were identified using MACS software (version 2.1.0). Consensus Motifs were identified using MEME with all significant peaks as input. TF target genes were annotated by peaks locating in gene promoters (upstream of gene start site or overlapping with gene start site).<br><br>2. RNA-seq analysis<br>RNA-seq reads were mapped to the P. aeruginosa genomes (NC_002516) using STAR, only the uniquely mapped reads were kept for the subsequent analyses. Differentially expressed genes were identified using DESeq2 (P-value < 0.05 and log2 Fold Change < -1 or log2 Fold Change > 1) 82. All the experiment has two repeats.<br><br>3. Network construction<br>For transcription factors with ChIP-seq and mutant RNA-seq data generated from our lab or known differentially expressed genes from previous studies, the transcriptional targets that are differentially expressed were identified as functional targets (Regulons). The integration and construction of regulatory network is performed in R (3.5.0). Master regulator analyses for QS, T3SS and T6SS were performed identified using Hypergeometric test in R (3.5.0). R package RedeR was used to visualize the network.<br><br>All code for data analysis is in "https://github.com/CityUHK-CompBio/PAGnet_code"

For manuscripts utilizing custom algorithms or software that are central to the research but not yet described in published literature, software must be made available to editors/reviewers. We strongly encourage code deposition in a community repository (e.g. GitHub). See the Nature Research guidelines for submitting code & software for further information.

Data

Policy information about availability of data

All manuscripts must include a data availability statement. This statement should provide the following information, where applicable:

- Accession codes, unique identifiers, or web links for publicly available datasets
- A list of figures that have associated raw data
- A description of any restrictions on data availability

ChIP-seq and RNA-seq data are available in the National Center for Biotechnology Information Gene Expression Omnibus under series GSE121243 and GSE128430.

Field-specific reporting

Please select the one below that is the best fit for your research. If you are not sure, read the appropriate sections before making your selection.

☒ Life sciences ☐ Behavioural & social sciences ☐ Ecological, evolutionary & environmental sciences

For a reference copy of the document with all sections, see nature.com/documents/nr-reporting-summary-flat.pdf

Life sciences study design

All studies must disclose on these points even when the disclosure is negative.

Sample size	No sample -size calculation were performed.
Data exclusions	No data were excluded from the analyses.
Replication	Comparing predicted regulatory relationships with RT-qPCR validations, PAGnet yielded a good performance (accuracy: 85.7%, precision: 84.4% and recall: 100%)
Randomization	There was no relevant randomization in this study.
Blinding	There was no relevant blindid in this study.

Reporting for specific materials, systems and methods

We require information from authors about some types of materials, experimental systems and methods used in many studies. Here, indicate whether each material, system or method listed is relevant to your study. If you are not sure if a list item applies to your research, read the appropriate section before selecting a response.

Materials & experimental systems

n/a	Involved in the study	
☐	☒	Antibodies
☒	☐	Eukaryotic cell lines
☒	☐	Palaeontology
☒	☐	Animals and other organisms
☒	☐	Human research participants
☒	☐	Clinical data

Methods

n/a	Involved in the study	
☐	☒	ChIP-seq
☒	☐	Flow cytometry
☒	☐	MRI-based neuroimaging

Antibodies

Antibodies used	Agarose-conjugated anti-VSV antibodies (Sigma)
Validation	https://www.sigmaaldrich.com/catalog/product/sigma/a1970?lang=en&region=HK

ChIP-seq

Data deposition

☒ Confirm that both raw and final processed data have been deposited in a public database such as GEO.

☒ Confirm that you have deposited or provided access to graph files (e.g. BED files) for the called peaks.

Data access links
May remain private before publication.

https://www.ncbi.nlm.nih.gov/geo/query/acc.cgi?acc=GSE121243
https://www.ncbi.nlm.nih.gov/geo/query/acc.cgi?acc=GSE128430

Files in database submission

mvfR1 ChIP-seq, mvfR2 ChIP-seq, soxR1 ChIP-seq, soxR2 ChIP-seq, rpoN1 ChIP-seq, rpoN2 ChIP-seq, Vfr1 ChIP-seq, Vfr2 ChIP-seq, algR1 ChIP-seq, algR2 ChIP-seq, exsA1 ChIP-seq, exsA2 ChIP-seq, gacA1 ChIP-seq, gacA2 ChIP-seq, 2588-1 ChIP-seq, 2588-2 ChIP-seq, mexT1 ChIP-seq, mexT2 ChIP-seq, FleQ1 ChIP-seq, FleQ2 ChIP-seq, GbdR1 ChIP-seq, GbdR2 ChIP-seq, PchR1 ChIP-seq, PchR2 ChIP-seq, PhoB1 ChIP-seq, PhoB2 ChIP-seq, SphR1 ChIP-seq, SphR2 ChIP-seq

Genome browser session
(e.g. UCSC)

http://archaea.ucsc.edu/cgi-bin/hgTracks?
hgS_doOtherUser=submit&hgS_otherUserName=huanghaobio&hgS_otherUserSessionName=pseuAeru

Methodology

Replicates

All the experiments have two repeats.

Sequencing depth

All ChIP-seq data is single-end, two repeats will be merged together for downstream analysis.
Input_rep1 ChIP-seq have 4911962 raw reads in total, 3724669 uniquely mapped reads, read length is 50. Input_rep2 have 4784701 raw reads in total, 3367353 uniquely mapped reads, read length is 50.
VqsM_rep1 ChIP-seq have 2511739 raw reads in total, 1633854 uniquely mapped reads, read length is 50. VqsM_rep2 have 4319882 raw reads in total, 2489915 uniquely mapped reads, read length is 50.
MvfR_rep1 ChIP-seq have 4316446 raw reads in total, 1254406 uniquely mapped reads, read length is 38. MvfR_rep2 have 4972117 raw reads in total, 1863385 uniquely mapped reads, read length is 38.
SoxR_rep1 ChIP-seq have 5100899 raw reads in total, 2330476 uniquely mapped reads, read length is 38. SoxR_rep2 have 5722076 raw reads in total, 2344985 uniquely mapped reads, read length is 38.
AlgR_rep1 ChIP-seq have 7442912 raw reads in total, 4791409 uniquely mapped reads, read length is 38. AlgR_rep2 have 4520418 raw reads in total, 3575959 uniquely mapped reads, read length is 38.
ExsA_rep1 ChIP-seq have 5124659 raw reads in total, 897428 uniquely mapped reads, read length is 38. ExsA_rep2 have 3840691 raw reads in total, 1347634 uniquely mapped reads, read length is 38.
GacA_rep1 ChIP-seq have 3873877 raw reads in total, 1368515 uniquely mapped reads, read length is 38. GacA_rep2 have 3906798 raw reads in total, 1269569 uniquely mapped reads, read length is 38.
CdpR_rep1 ChIP-seq have 4845991 raw reads in total, 2777438 uniquely mapped reads, read length is 38. CdpR_rep2 have 5167694 raw reads in total, 3119587 uniquely mapped reads, read length is 38.
MexT_rep1 ChIP-seq have 8031566 raw reads in total, 3906828 uniquely mapped reads, read length is 38. MexT_rep2 have 5167694 raw reads in total, 4456191 uniquely mapped reads, read length is 38.
RsaL_rep1 ChIP-seq have 5968264 raw reads in total, 5087155 uniquely mapped reads, read length is 38. RsaL_rep2 have 3393777 raw reads in total, 2875480 uniquely mapped reads, read length is 50.
FleQ_rep1 ChIP-seq have 2291781 raw reads in total, 1323295 uniquely mapped reads, read length is 150. FleQ_rep2 ChIP-seq have 1793512 reads in total, 1270576 uniquely mapped reads,read length is 150.
GbdR_rep1 ChIP-seq have 3486693 raw reads in total, 2845218 uniquely mapped reads, read length is 150. GbdR_rep2 ChIP-seq have 8308675 reads in total, 7242123 uniquely mapped reads,read length is 150.
PchR_rep1 ChIP-seq have 2679064 raw reads in total, 2042827 uniquely mapped reads, read length is 150. PchR_rep2 ChIP-seq have 7175715 reads in total, 6334657 uniquely mapped reads,read length is 150.
PhoB_rep1 ChIP-seq have 3478767 raw reads in total, 2870987 uniquely mapped reads, read length is 150. PhoB_rep2 ChIP-seq have 5888132 reads in total, 4868178 uniquely mapped reads,read length is 150.
SphR_rep1 ChIP-seq have 3419381 raw reads in total, 2432811 uniquely mapped reads, read length is 150. SphR_rep2 ChIP-seq have 6891114 reads in total, 5565852 uniquely mapped reads,read length is 150.

Antibodies

A1970 Sigma
Anti-VSV-Glycoprotein–Agarose antibody, Mouse monoclonal
clone P5D4, purified from hybridoma cell culture, PBS suspension

Peak calling parameters

macs2 callpeak -t algR.fastq.bam -c Input.fastq.bam -f BAM -g 6264404 --nomodel --extsize 75 -p 1e-5 -n algR

Data quality

Binding peaks (P < 1e-5) were identified using MACS software (version 2.1.0). For AlgR ChIP-seq data, we identified 620 narrowpeaks (P < 1e-5), all 620 peaks fulfill FDR < 0.05, and 577 peaks have more than 5-fold enrichment. For CdpR ChIP-seq data, we identified 47 narrowpeaks (P < 1e-5), all 47 peaks fulfill FDR < 0.05 but less than 5-fold enrichment. For ExsA ChIP-seq data, we identified 37narrowpeaks (P < 1e-5), all 37 peaks fulfill FDR < 0.05, and 6 peaks have more than 5-fold enrichment. For GacA ChIP-seq data, we identified 1125narrowpeaks (P < 1e-5), all 1125 peaks fulfill FDR < 0.05 but less than 5-fold enrichment. For MexT ChIP-seq data, we identified 13 narrowpeaks (P < 1e-5), all 13 peaks fulfill FDR < 0.05 but less than 5-fold enrichment. For MvfR ChIP-seq data, we identified 221 narrowpeaks (P < 1e-5), all 221 peaks fulfill FDR < 0.05 but less than 5-fold enrichment. For RpoN ChIP-seq data, we identified 1852 narrowpeaks (P < 1e-5), all 1852 peaks fulfill FDR < 0.05 but less than 5-fold enrichment. For RsaL ChIP-seq data, we identified 51 narrowpeaks (P < 1e-5), all 51 peaks fulfill FDR < 0.05 but less than 5-fold enrichment. For SoxR ChIP-seq data, we identified 230 narrowpeaks (P < 1e-5), all 230 peaks fulfill FDR < 0.05 but less than 5-fold enrichment. For Vfr ChIP-seq data, we identified 253 narrowpeaks (P < 1e-5), all 253 peaks fulfill FDR < 0.05 but less than 5-fold enrichment. For VqsM ChIP-seq data, we identified 104 narrowpeaks (P < 1e-5), all 104 peaks fulfill FDR < 0.05 but less than 5-fold enrichment. For FleQ ChIP-seq data, we identified 190 narrowpeaks (P < 1e-5), all 190 peaks fulfill FDR < 0.05, 155 peaks have less than 5-fold enrichment. For GbdR ChIP-seq data, we identified 93 narrowpeaks (P < 1e-5), all 93 peaks fulfill FDR < 0.05 but less than 5-fold enrichment. For PchR ChIP-seq data, we identified 134 narrowpeaks (P < 1e-5), all 98 peaks fulfill FDR < 0.05 but less than 5-fold enrichment. For PhoB ChIP-seq data, we identified 94 narrowpeaks (P < 1e-5), all 94 peaks fulfill FDR < 0.05 but less than 5-fold enrichment. For SphR ChIP-seq data, we identified 190 narrowpeaks (P < 1e-5), all 188 peaks fulfill FDR < 0.05 but less than 5-fold enrichment.

Software

ChIP-seq reads were mapped to the P. aeruginosa genomes (NC_002516) using Bowtie (Version 1.2.1.1). Only the uniquely mapped reads were kept for the subsequent analyses. All the experiments have two repeats and repeats were merged together for downstream analysis.Binding peaks (P < 1e-5) were identified using MACS software (version 2.1.0). Consensus Motifs were identified using MEME with all significant peaks as input. TF target genes were annotated by peaks locating in gene promoters (upstream of gene start site or overlapping with gene start site).
All code is in https://github.com/CityUHK-CompBio/PAGnet_code.
